# Transcriptomic and Proteomic Analyses of *Nepenthes ampullaria* and *Nepenthes rafflesiana* Reveal Parental Molecular Expression in the Pitchers of Their Hybrid, *Nepenthes* × *hookeriana*

**DOI:** 10.3389/fpls.2020.625507

**Published:** 2021-01-20

**Authors:** Muhammad Mu’izzuddin Zulkapli, Nur Syatila Ab Ghani, Tiew Yik Ting, Wan Mohd Aizat, Hoe-Han Goh

**Affiliations:** Institute of Systems Biology, Universiti Kebangsaan Malaysia, Bangi, Malaysia

**Keywords:** carnivory, hybrid, proteomics, transcriptomics, *Nepenthes*, pitcher fluid

## Abstract

*Nepenthes* is a genus comprising carnivorous tropical pitcher plants that have evolved trapping organs at the tip of their leaves for nutrient acquisition from insect trapping. Recent studies have applied proteomics approaches to identify proteins in the pitcher fluids for better understanding the carnivory mechanism, but protein identification is hindered by limited species-specific transcriptomes for *Nepenthes*. In this study, the proteomics informed by transcriptomics (PIT) approach was utilized to identify and compare proteins in the pitcher fluids of *Nepenthes ampullaria*, *Nepenthes rafflesiana*, and their hybrid *Nepenthes* × *hookeriana* through PacBio isoform sequencing (Iso-Seq) and liquid chromatography-mass spectrometry (LC-MS) proteomic profiling. We generated full-length transcriptomes from all three species of 80,791 consensus isoforms with an average length of 1,692 bp as a reference for protein identification. The comparative analysis found that transcripts and proteins identified in the hybrid *N.* × *hookeriana* were more resembling *N. rafflesiana*, both of which are insectivorous compared with omnivorous *N. ampullaria* that can derive nutrients from leaf litters. Previously reported hydrolytic proteins were detected, including proteases, glucanases, chitinases, phosphatases, nucleases, peroxidases, lipid transfer protein, thaumatin-like protein, pathogenesis-related protein, and disease resistance proteins. Many new proteins with diverse predicted functions were also identified, such as amylase, invertase, catalase, kinases, ligases, synthases, esterases, transferases, transporters, and transcription factors. Despite the discovery of a few unique enzymes in *N. ampullaria*, we found no strong evidence of adaptive evolution to produce endogenous enzymes for the breakdown of leaf litter. A more complete picture of digestive fluid protein composition in this study provides important insights on the molecular physiology of pitchers and carnivory mechanism of *Nepenthes* species with distinct dietary habits.

## Introduction

*Nepenthes* comprises unique carnivorous tropical plants with pitcher organs at the ends of leaf tips for the capture, digestion, and absorption of insects to grow in nutrient-poor soil. There are more than 150 *Nepenthes* species geographically distributed in Southeast Asia, including Borneo, Philippines, and Sumatra (Murphy et al., 2020). The species diversification of this genus in their pitcher morphological features, ecology, and nutrient acquisition attracted many evolutionary studies of *Nepenthes* (Clarke and Moran, 2016).

*Nepenthes* species are mostly insectivorous but previous studies showed that *Nepenthes ampullaria*, which is predominantly found in the heath and swamp forests compared to open habitats like other species, possesses detritivore traits to trap leaf litter as a nutrient source (Moran et al., 2003). This genus is also well-known for natural and artificial hybridization. One of the common natural hybrids is *Nepenthes* × *hookeriana*, between *N. ampullaria* and *Nepenthes rafflesiana*. This hybridization was initially identified based on their common morphological characters (Clarke and Wong, 1997; Clarke, 2001) and later verified through protein and genetic marker analyses (Yulita and Mansur, 2012; Biteau et al., 2013), which also suggested a closer relationship between *N.* × *hookeriana* and *N. rafflesiana* than *N. ampullaria*. To date, there is no comprehensive report comparing the molecular expression in the pitchers and pitcher fluids of the hybrid and *N. rafflesiana* as carnivores, with *N. ampullaria* being an omnivore. This gap of knowledge was pointed out by Pavlovič (2012) on the adaptive radiation of *Nepenthes* nutrient sequestration strategies.

The pitchers with acidic fluids and secreted enzymes are important for trapping and digesting invertebrate prey (Ravee et al., 2018; Gilbert et al., 2020). Several digestive enzymes are commonly reported to be secreted into the pitcher fluids, which include aspartic proteases, nucleases, and pathogenesis-related (PR) proteins (Athauda et al., 2004; Hatano and Hamada, 2012; Buch et al., 2014; Rottloff et al., 2016; Fukushima et al., 2017). In comparison, knowledge of nutrient uptake and transportation is more limited. Furthermore, the regulatory mechanism of protein secretion and replenishment remains poorly understood (Wan Zakaria et al., 2016b, 2019; Goh et al., 2020). Protein identification in the pitcher fluids is limited by the unusual amino acid composition and the limited sequence information for *Nepenthes* (Lee et al., 2016). There are only 760 UniProtKB entries for the taxonomy *Nepenthes* as of August 2020, the majority of which are the maturase K and ribosomal protein sequences apart from those digestive enzymes mentioned above.

Species-specific transcriptome sequences are ideal for protein identification. Hence, we applied proteomics informed by transcriptomics (PIT) approach in this study to compare protein profiles of the hybrid *N.* × *hookeriana* with its parents *N. rafflesiana* and *N. ampullaria* for comparative protein profile analysis to elucidate the pitcher fluid protein composition of each species. Comparing the molecular profiles among the three species not only can explore the differences in fluid protein composition due to dietary habits but also validate their relationship. Furthermore, this study provides a reference list of endogenous proteins secreted upon pitcher opening for further studies on the regulation of secreted proteins into the pitcher fluids.

## Materials and Methods

### Plant Materials

*Nepenthes* plants (*N. ampullaria*, *N. rafflesiana*, and *N.* × *hookeriana*) originated from the Endau Rompin National Park Malaysia were grown in a common garden at the experimental terrace (2°55′11.5″N 101°47′01.4″E) of Universiti Kebangsaan Malaysia under natural weather conditions. Developing pitchers were monitored daily and covered with mesh nets to prevent insect entry. Newly opened pitchers within 24 h of lid opening were harvested between June to August 2015 in the morning 9:00–10:00 am (Zulkapli et al., 2017). All of the fluids from individual pitchers were poured into separate Falcon tubes, while the whole pitcher tissues excluding the tendril were kept in separate plastic bags and frozen in liquid nitrogen before stored at −80°C.

### PacBio Isoform Sequencing

Total RNA was extracted from pitcher tissues using the modified cetyltrimethylammonium bromide (CTAB) protocol (Abdul-Rahman et al., 2017). The quality and integrity of extracted total RNA were determined using Nanodrop ND-1000 (Thermo Fisher Scientific Inc., United States) and Agilent 2,100 bioanalyzer (Agilent Technologies, United States), respectively. Total RNA with RNA integrity number (RIN) >8 was submitted for library preparation and sequencing at Icahn Medical Institute (Mount Sinai, New York City, United States). One replicate per species with the highest RIN was chosen for sequencing. Full-length cDNAs were prepared using SMARTer PCR cDNA synthesis kit (Clontech) according to the manufacturer’s protocols. Double-stranded cDNAs were subjected to size selection using BluePippin (Sage Science, MA, United States) at the MW range of 1–3 kb. The PCR amplification profile was 95°C for 2 min, 15 cycles × (98°C for 20 s, 65°C for 15 s, 72°C for 4 min), and a final extension at 72°C for 5 min. Due to low yield after selection, *N. ampullaria* sample was further amplified for nine cycles (98°C for 20 s, 65°C for 15 s, 72°C for 1 min 45 s) and was size-selected again before preparing the SMRTbell library with a minimum of 1 μg of dsDNA based on the manufacturer’s SMRTbell template protocol (SMRTbell Template Preparation Kit 1.0). The SMRTbell libraries were purified by two sequential 0.45 × AMPure PB purifications (Pacific Biosciences) after exonuclease digestion of incomplete SMRTbell templates. Libraries were quantified by fluorimetry and assayed for quantity and size distribution by Bioanalyzer. A single SMRTbell library for individual species was sequenced using SMRT Cell v3 with P6-C4 chemistry on the PacBio RS II platform (Pacific Biosciences), each at a concentration of 110 pM (Zulkapli et al., 2017).

Subreads <300-bp and reads with quality <0.75 (corresponding to a predicted error rate of >25%) were filtered out. Sub-reads were filtered and subjected to circular consensus sequence (CCS) read analysis using PacBio SMRT Analysis Server v2.3.0 following the RS_IsoSeq protocol. In brief, cDNA primer and poly-A tails were removed and the read of inserts (ROIs) were classified into full-length and non-full-length. Iterative clustering for error correction (ICE) algorithm was also used and quiver polishing was performed to generate consensus isoform sequences at a high QV value of 0.99 and expected size of 1–2 kb. For the reference transcriptome, raw reads from all three species were combined for the CCS read analysis.

Raw sequences were deposited into the Sequence Read Archive (SRA) under BioProject PRJNA299862 with the following identifiers: SRX2692198 (*N. ampullaria*), SRX2692197 (*N. rafflesiana*), and SRX2692196 (*N.* × *hookeriana*) (Zulkapli et al., 2017). Consensus isoform sequences can be accessed from the TSA repository: GGLJ00000000.1 (*N. ampullaria*), GGLG00000000.1 (*N. rafflesiana*), and GGLF00000000.1 (*N.* × *hookeriana*).

### Transcriptomics Analysis

Bioinformatics methods were applied to analyze the consensus isoform sequences aiming to compare the transcripts between the three *Nepenthes* species, including BLAST, TransDecoder, Trinotate, OrthoVenn, WEGO, and KAAS. Consensus isoform sequences of the hybrid *N.* × *hookeriana* were searched against the consensus isoform sequences of *N. ampullaria* and *N. rafflesiana* using local BLASTN v.2.6.0 with an *E*-value cut-off of 1e^–5^.

Trinotate (Bryant et al., 2017) was used for functional annotation of individual transcriptomes based on different methods that include homology search (BLAST+/UniProt), protein domain identification (HMMER/Pfam), prediction of signal peptide (SignalP), and transmembrane domain (TmHMM), as well as searches against eggNOG (evolutionary genealogy of genes: Non-supervised Orthologous Groups), Gene Ontology (GO), and Kyoto Encyclopedia of Genes and Genomes (KEGG) databases.

The GO annotations from Trinotate report were plotted using Web Gene Ontology Annotation (WEGO) (Tyanova et al., 2016), with further selection on the GO groups related to four unique physiology of carnivorous plants, namely trapping, digestion, absorption, and defense. The functional annotation in KEGG GENES database was obtained using KEGG Automatic Annotation Server (KAAS) that assigns KEGG Orthology (KO) to KEGG pathways given a set of protein sequences as input (Moriya et al., 2007). Venn diagram analysis was performed using Venny version 2.1.0 (Oliveros, 2007-2015).

Protein-coding sequences (CDS) predicted from the consensus isoform sequences using TransDecoder (Haas et al., 2013) were used as a reference dataset for protein identification and comparative analysis using OrthoVenn2 (Xu et al., 2019) with default parameters: *E*-value cutoff of 1e^–5^ for all-to-all similarity comparisons and the inflation value of 1.5 for the generation of orthologous clusters using the Markov Cluster Algorithm. The reference predicted protein sequences^1^ were further annotated using eggNOG 5.0 (Huerta-Cepas et al., 2019) for functional categorization using eMapper v2.0^2^ with default settings. Overrepresentation and underrepresentation analyses of KEGG pathway and eggNOG were performed using the hypergeometric test function in MS Excel.

### Protein Extraction and Processing

Pitcher fluids were filtered through 25 mm acrodisc syringe filter with PVDF membrane of 0.2 μm pore size (Pall, United States) pre-conditioned using 1 mL UHP water (Mili-Q). Proteins were then concentrated by ultrafiltration through a Microsep Advance Centrifugal Devices with Omega membrane (Pall, United States) at a molecular weight cut-off of 10 kDa, rinsed with 1 mL of UHP water. Supernatants (>10 kDa) were collected and further concentrated to 100 μL through speed vacuum (Wan Zakaria et al., 2018). Aliquots of 20 μL were used for sodium dodecyl sulfate-polyacrylamide gel electrophoresis (SDS-PAGE) and the remaining 80 μL were pooled together from nine biological replicates for LC-MS/MS analysis.

Sodium dodecyl sulfate-polyacrylamide gel electrophoresis was performed using Bio-Rad electrophoresis apparatus (Bio-Rad, United States). Loading buffer (0.2 M Tris–HCl [pH 6.8], 10% SDS, 20% glycerol, 10 mM β-mercaptoethanol, 1% bromophenol blue, and water) was added to the protein sample and heated at 95°C for 10 min to break down the disulfide bonds. Two gel layers were prepared, namely the stacking gel with bis-acrylamide concentration of 12.5% (pH 8.8) and the separating gel with bis-acrylamide concentration of 4% (pH 6.8). The electrophoresis was performed at 75 V for 25 min followed by 125 V for 90 min. The MS-incompatible silver staining method was performed (Wan Zakaria et al., 2019). Gels were fixed in fixation solution with 30% ethanol and 10% acetic acid overnight before washed thoroughly in 30% ethanol for 20 min and soaked in distilled water for 20 min followed by sensitivity enhancing solution containing 8 mM sodium thiosulfate pentahydrate. The gels were washed thrice with distilled water and soaked in silver stain solution containing 11 mM silver nitrate and 0.15% formaldehyde. Then, the gels were washed thrice and soaked in the development solution with 0.5 M sodium carbonate and 0.2% formaldehyde. Once protein strips appeared, the reaction was stopped by washing the gels with stop solution containing 0.5 mM EDTA for 10 min. Gels were rinsed with distilled water, analyzed, and captured via Densitometer accompanied with Quantity One version 4.6.7 (Bio-Rad, United States). The protein band sizes were estimated using the BLUEstain protein ladder (11–245 kDa) (GoldBio).

### Proteomics Analysis of Pitcher Fluids Using nanoLC-MS/MS

For gel-free liquid chromatography tandem mass spectrometry (LC-MS/MS) analysis, solid phase extraction (SPE) was performed using the 1 cc Oasis HLB cartridges containing the Oasis HLB sorbent (OASIS, United States). SPE eluent was dried through speed vacuum and was rehydrated by 170 μL of 0.5% formic acid with 20 μL aliquot for SDS-PAGE and the remaining 150 μL was used for in-solution digestion. Trypsin digestion was conducted at a ratio of 1:100 (Wan Zakaria et al., 2018). Digested sample dried through speed vacuum and rehydrated by 35 μL of 0.5% formic acid with 3 μL aliquot for SDS-PAGE and the remaining was used for Zip-Tip protocol. Zip-Tip protocol was performed using Thermo Scientific Pierce C18 Tips (Thermo, United States) prior to MS analysis. Sample was speed vacuum and rehydrated with 45 μL of 0.1% formic acid prior to LC-MS/MS analysis.

All experiments were performed in a nanoflow LC system, Easy-nLC (Thermo) equipped with EASY-Spray Column Acclaim PepMap C18 and coupled (Thermo) to Orbitrap Fusion Tribrid mass spectrometer (Thermo, United States). Protein samples were loaded onto the pre-column and the peptides were analyzed using linear-gradient program with flow rate of 300 nL/min for 0.1% formic acid (solution A) and 0.1% formic acid in acetonitrile (solution B) and gradients were set as followed: (i) 5–40% B for 91 min, (ii) 95% B for 2 min, (iii) 95% B for 6 min, and (iv) 5% B for 2 min. Each pooled sample from nine biological replicates was injected three times in two independent analyses for data collection resulting in six spectra for each species.

MS/MS data were retrieved using mass spectrometer LTQ Orbitrap XL (Thermo Scientific). Full scan profile mass spectra (OTMS1) was obtained using the following parameters in top speed mode: scan range (*m/z*) = 201–1800 Da; cycle time = 3 s; resolution = 120,000; AGC target = 4.0e^5^; maximum injection time = 50 ms; precursor selection with charge state of 2–7; dynamic exclusion duration = 20 s; intensity = 5000. The parameters used for MSMS (ITMS2) analyses were as followed: rapid scan rate; CID NCE = 30%; HCD NCE = 28%; isolation window = 1.6 m/z; AGC target = 1.0e^2^; maximum injection time = 250 ms. Raw data, sequence files, and results were deposited to the ProteomeXchange Consortium via the PRIDE partner repository (Perez-Riverol et al., 2019) with data set identifier PXD007599.

### Protein Identification

Liquid chromatography tandem mass spectrometry raw files were processed for peptide identification using MaxQuant version 1.5.3.30 (Tyanova et al., 2016) through peptide to spectrum matching (PSM) pipeline using three digestion (Trypsin/P) settings: specific, semispecific, and unspecific. Carbamidomethylation and methionine oxidation were used as fixed and variable modifications. The MS/MS spectrum was searched through Andromeda searching engine (Cox et al., 2011) integrated with MaxQuant, against the reference data set of predicted proteins obtained from TransDecoder, in addition to 55 previously reported protein sequences (Lee et al., 2016; Rottloff et al., 2016) with 358 potential contaminants and reversed sequences. Initial mass tolerance was set to 4.5, 20 ppm on MS, and 0.5 Da on MS/MS. For peptide identification, the false discovery rate was set to 0.01, minimum peptide length was 7 amino acids and the maximum mis-cleavages allowed were 2. For peptide quantification, MaxLFQ algorithm (Cox et al., 2014) was used based on default parameters with minimum ratio count set to 1. For peptide matching, the retention time window was set to 30 s. Proteins with more than one peptide or one peptide with at least one MS/MS and intensity values greater than 0 were considered identified and present. Proteins obtained from MaxQuant identification were used for sequence comparisons using constraint-based alignment tool (COBALT) (Papadopoulos and Agarwala, 2007) and Clustal Omega (Sievers and Higgins, 2018).

## Results

Transcriptomics and proteomics analyses of *N. ampullaria, N. rafflesiana*, and their hybrid, *N.* × *hookeriana* were conducted with an overview of the methods illustrated in Supplementary Figure 1.

### Transcriptome Profiling of *Nepenthes* through PacBio Isoform Sequencing

The transcriptome libraries of individual *Nepenthes* species were generated using PacBio isoform sequencing (Iso-Seq). A total of 26,130, 30,558, and 33,279 consensus isoforms were generated for *N. ampullaria*, *N. rafflesiana*, and *N.* × *hookeriana*, respectively, with an average length of 1,625, 1,680, and 1,722 bp (Supplementary Table 1). The three transcriptomes from individual *Nepenthes* species were combined to form a reference transcriptome containing a total of 80,791 consensus isoforms with an average length of 1,692 bp. Despite having the lowest number of read of insert (ROI), the hybrid expressed the highest number of consensus isoforms, indicating more varied transcripts in the hybrid. The local BLASTN search of the hybrid consensus isoforms against the parents found more hits with *N. rafllesiana* (93.2%) than *N. ampullaria* (89.2%) at similarity >60%, which suggests more similar transcriptome sequences between the hybrid and *N. rafllesiana* (Supplementary Figure 2).

Functional annotation for individual *Nepenthes* transcriptomes and the reference was performed through the Trinotate pipeline using transcript and predicted peptide sequences (Table 1 and Supplementary File 1). Homology searches of reference transcriptome performed using BLASTX and BLASTP found 53,917 (66.7%) hits to UniProt, 34,524 (42.7%) hits to Pfam, 22,560 (27.9%) hits to eggNOG, 37,816 (46.8%) hits with KO, and 58,635 (72.5%) hits to Arabidopsis genes. In total, 64,455 (79.8%) consensus isoforms of the reference transcriptome were functionally annotated with at least one database. Furthermore, predictions of signal peptides (SignalP) and transmembrane helices (TmHMM) annotated 3,121 (3.9%) and 10,510 (13.0%) peptides, respectively.

**TABLE 1:** Functional annotation of *Nepenthes* transcriptomes using Trinotate pipeline.

**Number**	***N. ampullaria***	***N. rafflesiana***	***N.* × *hookeriana***	**References**
Consensus isoform	26,130	30,558	33,279	80,791
Protein (CDS)	19,463	26,677	30,096	65,757
**Functional annotation**				
BLASTX	16,540	21,615	23,587	53,440
BLASTP	11,139	15,094	17,176	37,078
BLASTX/P	16,664	21,814	23,768	53,917
GO_BLAST	15,198	19,782	21,726	48,887
Pfam	10,455	14,192	16,103	34,524
GO_Pfam	7,836	10,408	12,107	25,561
eggNOG	6,984	9,045	9,896	22,560
SignalP	918	1,265	1,500	3,121
TmHMM	3,150	4,219	4,990	10,510
KO	11,785	15,170	16,553	37,816
AGI	18,258	23,491	25,696	58,535

*BLASTX, hits of isoform sequences to UniProt database; BLASTP, hits of predicted protein sequences to UniProt database; BLASTX/P, combined hits from BLASTX and BLASTP; GO_BLAST, GO annotation from BLAST; Pfam, hits to Pfam database; GO_Pfam, GO annotation from Pfam; eggNOG, hits to orthologous genes in eggNOG database; SignalP, prediction of signal peptide; TmHMM, prediction of transmembrane protein; KO, KEGG Orthology from KAAS; AGI, BLASTN hits to Arabidopsis genes.*

**FIGURE 1:**
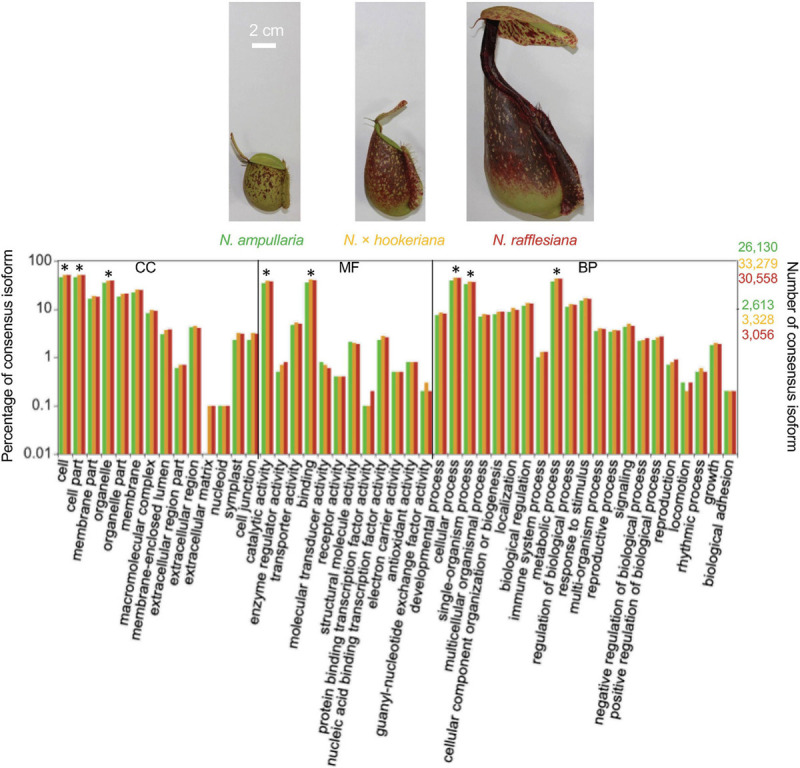
The distribution of Gene Ontology (GO) terms of annotated consensus isoforms from all three *Nepenthes* species using WEGO based on cellular component (CC), molecular function (MF), and biological process (BP). Asterisks (^∗^) represent significant (*P* < 0.001) differences in Pearson Chi-Square test. The bar chart is color-coded according to the font colors. Photos of the pitcher samples from the three species are displayed above with the same scale.

The annotated transcriptomes of the three *Nepenthes* species were assigned with 495 GO terms according to three main GO categories: biological process, molecular function, and cellular component with 310, 100, and 85 terms, respectively, (Supplementary File 2 and Supplementary Table 2). The distribution of GO annotation was visualized in WEGO analysis (Figure 1). The top terms for cellular component were cell, cell part, and organelle; for molecular function were the catalytic activity and binding; while for biological process were cellular process, metabolic process, and single-organism process, which showed significant differences between the three species. The analysis showed more significant differences of GO annotations between the two parent species (*N. ampullaria vs*. *N. rafllesiana*, 15.4%) than between the hybrid and *N. rafllesiana* (8.7%) compared to *N. ampullaria* (12.3%), indicating more similar functional genes between the hybrid and *N. rafllesiana* (Supplementary Table 2).

Meanwhile, KEGG annotation using KAAS against the KEGG GENES database found hits to 2,432, 2,846, and 2,663 KO, which mapped to 395, 398, and 396 KEGG pathways for *N. ampullaria, N. rafflesiana*, and *N.* × *hookeriana*, respectively, (Supplementary File 2 and Supplementary Table 3). Majority (392) of the KEGG pathways were common in all three species. Two KEGG pathways, mucin type O-glycan biosynthesis and glycosaminoglycan biosynthesis-keratan sulfate with beta-galactoside alpha-2,3-sialyltransferase (K00780), were unique to *N. ampullaria*. A protein O-mannose beta-1,4-N-acetylglucosaminyltransferase for mannose type O-glycan biosynthesis (K18207) was unique to *N*. *rafflesiana*; while a 2,4-dihydroxy-1,4-benzoxazin-3-one-glucoside dioxygenase (K13229) for benzoxazinoid biosynthesis was unique in the hybrid. Meanwhile, a UDP-N-acetylglucosamine acyltransferase (K00677) in cationic antimicrobial peptide (CAMP) resistance was found only in the parents (*N. ampullaria* and *N. rafflesiana*). Three pathways shared between the hybrid and *N*. *rafflesiana* were absent in *N. ampullaria*: glycosphingolipid biosynthesis - lacto and neolacto series with a lactosylceramide 4-alpha-galactosyltransferase (K01988), caprolactam degradation with an alcohol dehydrogenase (NADP+) (K00002), and a crocetin glucosyltransferase (K21371) for the biosynthesis of various secondary metabolites—part 1.

**FIGURE 2:**
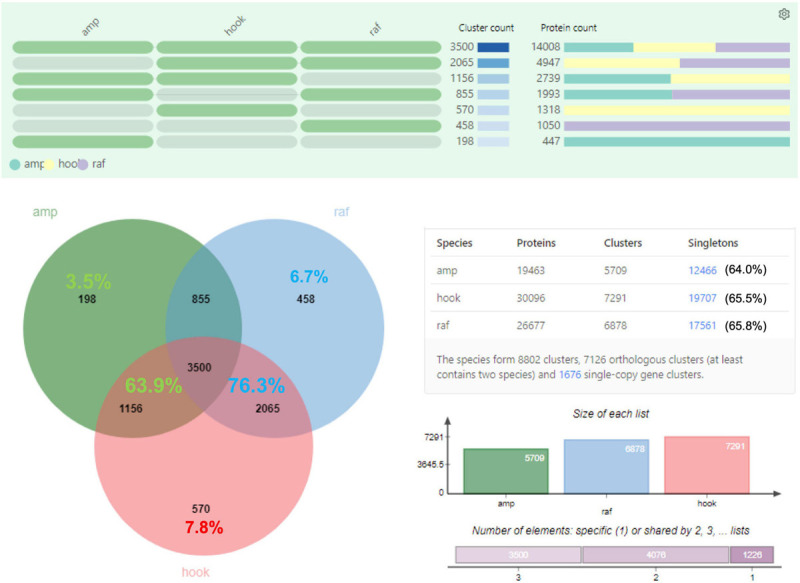
OrthoVenn analysis on the distribution of orthologous protein clusters of predicted protein sequences. Top part shows the cluster and protein counts in different sections of the Venn diagram, which shows the number of protein clusters. Percentages in the shared regions of Venn diagram between the hybrid and parents are calculated based on the total clusters in the hybrid; the percentages in the non-shared sections are based on respective species. The table on the lower right summarizes the numbers of proteins, clusters, and singletons in each species. Singletons are protein sequences that do not form any cluster with other sequences and are shown with percentages out of total number of protein sequences in the respective species. The charts on the lower right show the size of protein clusters in each species and the cumulative numbers of shared elements based on the Venn diagram. amp: *N*. *ampullaria*; raf: *N*. *rafflesiana*; hook: *N*. × *hookeriana*.

Transdecoder analysis predicted a total of 19,463, 26,677, 30,096, and 65,757 protein-CDS from 14,523, 19,683, 22,192, and 48,663 consensus isoforms, respectively, for *N. ampullaria*, *N. rafflesiana, N.* × *hookeriana*, and reference (Table 1 and Supplementary Table 4). A total of 53,235 (83.5%) predicted protein sequences of the reference can be functionally categorized by eggNOG 5.0 mapper (Supplementary File 1). Comparative analysis by OrthoVenn using predicted protein sequences identified 3,500 orthologous protein clusters shared among the three *Nepenthes* species in which 1,676 were single-copy gene clusters with ∼65% singletons without orthologs among the species (Figure 2 and Supplementary Table 4). The higher number of clusters shared between the hybrid and *N. rafflesiana* (76.3%) than with *N. ampullaria* (63.9%) corroborates the results from BLASTN analysis that reflects a closer genetic relationship between the hybrid and *N. rafflesiana* based on our samples in this study.

### Proteomic Profiling of *Nepenthes* Pitcher Fluids

**FIGURE 3:**
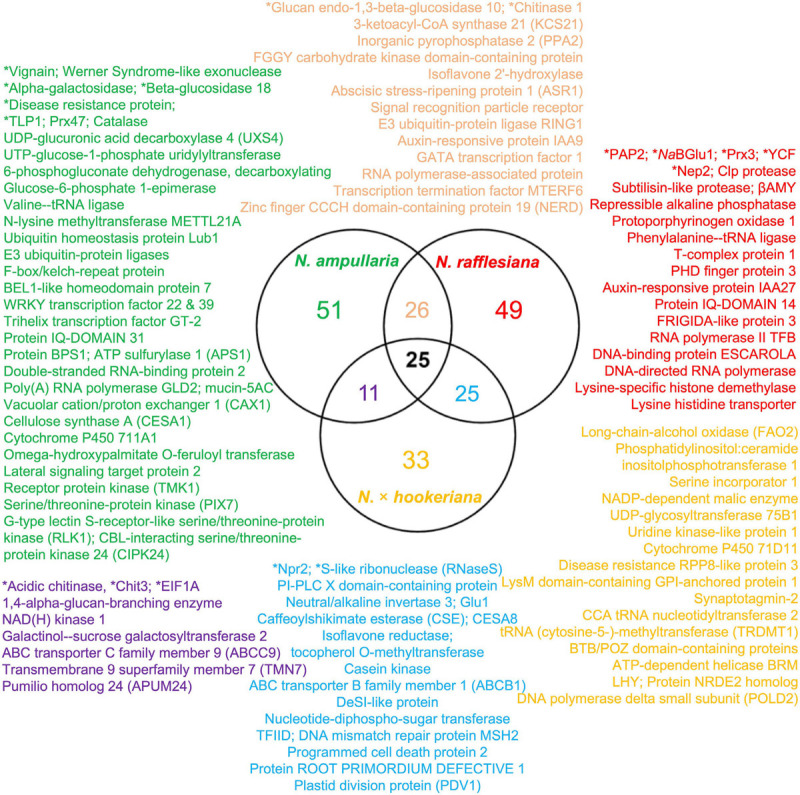
Venn diagram analysis of proteins identified from proteomics analysis. Selected proteins for each intersection are listed according to the font colors. Asterisks (^∗^) indicate proteins reported previously in the pitcher fluids of other *Nepenthes* species. Refer to Supplementary File 3 for details.

Proteins were extracted from nine biological replicates of pitcher fluids for each *Nepenthes* species and examined through SDS-PAGE analysis with silver staining at each step of sample processing (Supplementary Figure 3). These nine replicates were pooled for each species and analyzed using the nanoLC-MS/MS. MS data were processed using MaxQuant for searching against predicted protein sequences of the reference transcriptome and protein sequences from previous studies (Lee et al., 2016; Rottloff et al., 2016) through peptide spectrum matches (PSM). Proteins were identified using specific, semispecific, and unspecific digestion settings for a comprehensive analysis due to the hydrolytic proteins in the pitcher fluids with a possibility of non-specific digestion (Lee et al., 2016). The analysis identified a total of 220 proteins from *Nepenthes* pitcher fluids: 125 in *N. rafflesiana*, 113 in *N. ampullaria*, and 94 in *N.* × *hookeriana* (Supplementary File 3). The least number of fluid proteins was identified in the hybrid despite having the highest number of transcriptomic predicted protein sequences (Table 1). According to the Venn diagram analysis (Figure 3), more proteins were shared between *N.* × *hookeriana* and *N. rafflesiana* (50 proteins) than *N. ampullaria* (36 proteins), which is consistent with the OrthoVenn analysis. The number of unique proteins in *N. ampullaria* (51, 45%), *N. rafflesiana* (49, 39%), and *N.* × *hookeriana* (33, 35%) were proportionally higher than that of OrthoVenn analysis (3.5–7.8%, Figure 2). A total of 25 proteins were shared among the three species as listed in Table 2.

**TABLE 2:** List of 25 identified pitcher fluid proteins shared by *N. ampullaria*, *N. rafflesiana*, and *N.* × *hookeriana*.

**Biological process**	**Protein**	**Function**
Protein metabolism	Nepenthesin-1 (Nep1)	Aspartic protease
	Nepenthesin-3 (Nep3)	
	Nepenthesin-4 (Nep4)	
	Nepenthesin-5 (Nep5)	
	Neprosin-1 (Npr1)	Prolyl endoprotease
Lipid metabolism	Lipid phosphate phosphatase 2 (LPP2)	Lipid transfer
	Non-specific lipid transfer protein GPI-anchored 1 (LTPG1)	
Nucleic acid metabolism	Purple acid phosphatase (*Nr*PAP1)	Metallophosphatase
Polysaccharide metabolism	DOMON-like domain with heme-binding motif (*Nr*Dom1)	Glycoside hydrolase
Cell wall-related metabolism	Fasciclin-like arabinogalactan protein 7	Surface adhesion
	Fasciclin-like arabinogalactan protein 13	
	*NAC domain-containing protein 43 (NAC043)	Secondary cell wall biogenesis
Secondary metabolism	*12-oxophytodienoate reductase 3	Jasmonic acid biosynthesis
ROS regulation	Cation peroxidase 1 (*Nr*Prx1)	Peroxidase
	Cation peroxidase 1 (Prx1)	
	*Glutathione S-transferase (DHAR2)	ROS scavenging
Signal transduction	*Calmodulin-binding receptor-like cytoplasmic kinase 3 (CRCK3)	Serine family protein kinase
	*Serine/threonine protein phosphatase 2A 57 kDa regulatory subunit (B’KAPPA)	Phosphatase 2A regulatory subunit
Protein regulation	*F-box*/LRR-repeat protein At4g29420	Component of E3 ubiquitin ligase complex
Transporter	*Metal-nicotianamine transporter (YSL3)	Metal transporter
	*Probable sugar phosphate/phosphate translocator At3g11320	Carbohydrate transport
Gene regulation	*Polycomb protein Pcl-like	Transcription suppressor
	*RNA polymerase-associated protein C651.09c	Transcription elongation
Uncategorized	*Stress response protein NST1	
	*NADH:flavin oxidoreductase/NADH oxidase	

*Proteins not reported previously.

Based on the eggNOG 5.0 functional categorization of all proteins found in the pitcher fluids, “Translation, ribosomal structure and biogenesis,” “Transcription,” “Posttranslational modification, protein turnover, chaperones,” and “Function unknown” were found to be overrepresented (*P* < 0.05); “RNA processing and modification,” “Amino acid transport and metabolism,” and “Signal transduction mechanisms” were found to be underrepresented (*P* < 0.05) proportionally to the reference transcriptome (Supplementary File 3). There was no specific overrepresentation in individual species, except the hybrid with a disproportionally higher number of proteins with “Function unknown.”

**FIGURE 4:**
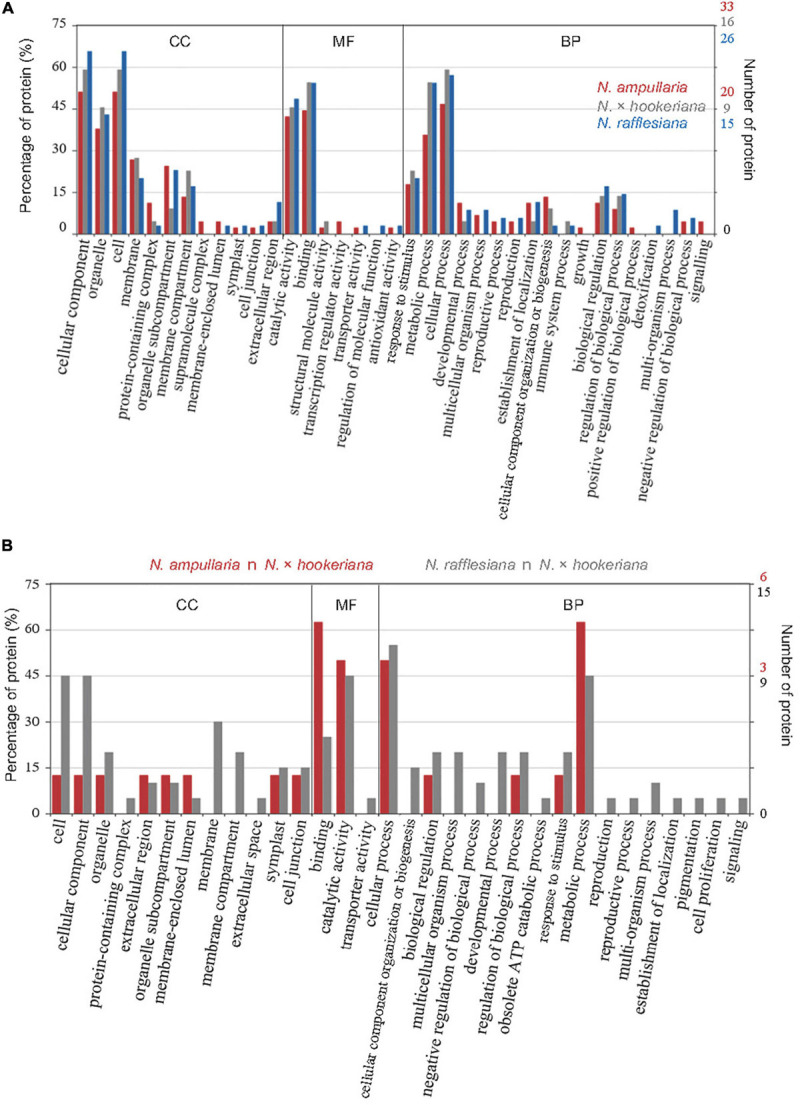
GO distribution of identified proteins from proteomics analysis based on cellular component (CC), molecular function (MF), and biological process (BP). **(A)** Distribution of GO terms shared among all *Nepenthes* species. **(B)** Distribution of GO terms shared between parent and hybrid species, that is, *N. ampullaria* vs. *N.* × *hookeriana* (red), and *N. rafflesiana* vs. *N.* × *hookeriana* (gray).

The distribution of GO annotation for shared and unique proteins in *Nepenthes* species was analyzed in WEGO (Figure 4A). The identified proteins from MaxQuant analysis were grouped into 18 biological processes, with eight biological processes common in all three species including cellular process, metabolic process, response to stimulus, biological regulation, cellular component organization or biogenesis, establishment of localization, and developmental process in the order of protein abundance. Two GO terms, catalytic activity (GO:0003824) and binding (GO:0005488), were annotated for more than 40% of proteins under the molecular function category, which indicates the predominant functions of proteins in the digestive pitcher fluids. Meanwhile, the comparison of GO annotation between the hybrid and parents found 17 common GO terms between the hybrid and *N. rafflesiana* in biological process compared to five with *N. ampullaria* (Figure 4B).

### Pitcher Fluid Proteins Related to Carnivory Traits of *Nepenthes*

To explore carnivory mechanism of the three *Nepenthes* species, we focused on proteins with GO terms related to physiological properties of carnivorous plants, namely trapping, digestion, nutrient absorption, and defense. The proteins annotated with “catalytic activity” are mainly hydrolases, oxidoreductases, and transferases comprising nepenthesins, neprosins, purple acid phosphatases, lipid phosphate phosphatase, S-like ribonuclease (RNaseS), glucosidases, glucanases, and peroxidases commonly reported in *Nepenthes* pitcher fluids (Table 3).

**TABLE 3:** List of identified endogenous proteins important in *Nepenthes* carnivory traits from non-fed pitchers.

Function	Protein	Presence^‡^			Reported in other species
		**A**	**H**	**R**	
Protein metabolism	Nepenthesin-1, Nep1	√	√	√	*N.* × *ventrata* (Lee et al., 2016); *N. alata* (Hatano and Hamada, 2008); *N. distillatoria*, *N. gracilis* (Athauda et al., 2004); *N. sanguinea* (Rottloff et al., 2016); and *N. mirabilis* (Buch et al., 2015)
	Nepenthesin-2, Nep2	−*	−*	√	
	Nepenthesin-3, Nep3	√	√	√	
	Nepenthesin-4, Nep4	√	√	√	
	Nepenthesin-5, Nep5	√	√	√	
	Neprosin-1, Npr1	√	√	√	*N.* × *ventrata* (Lee et al., 2016; Wan Zakaria et al., 2019)
	Neprosin-2, Npr2	−*	√	√	
	Vignain	√	−*	−*	*N. ventricosa* (Stephenson and Hogan, 2006)
Nucleic acid metabolism	S-like ribonuclease, *Nr*RNaseS	−	√	√	*N.* × *ventrata* (Lee et al., 2016; Wan Zakaria et al., 2019); *N. bicalcarata* (Stephenson and Hogan, 2006); and *N. ventricosa* (Nishimura et al., 2014)
Lipid metabolism	Non-specific lipid transfer protein GPI-anchored 1, LTPG1	√	√	√	*N. alata* (Hatano and Hamada, 2008); *N. mirabilis* (Rottloff et al., 2016)
	Lipid phosphate phosphatase 2, LPP2	√	√	√	
Metallo-phosphatase	Purple acid phosphatase, PAP1	√	√	√	*N.* × *ventrata* (Lee et al., 2016; Wan Zakaria et al., 2019)
Polysaccharide	β-1,3-glucanase, *Nr*Glu1	−	√	√	*N.* × *ventrata* (Lee et al., 2016)
metabolism/	β-1,3-glucanase, *Na*BGlu1	−	−	√	*N. alata* (Hatano and Hamada, 2008; Rottloff et al., 2016)
Defense response	Chitinase class III, Chit3 Chitinase class IV, Chit1	√ √	√ −	− √	*N.* × *ventrata* (Lee et al., 2016; Wan Zakaria et al., 2019); *N. alata* (Hatano and Hamada, 2008; Rottloff et al., 2011, 2016); *N. khasiana* (Eilenberg et al., 2006); *N. singalana*, *N. gracilis*, *N. mirabilis*, and *N. rafflesiana* (Rottloff et al., 2011)
	Thaumatin-like protein, TLP1	√	−*	−*	*N. alata* (Hatano and Hamada, 2008; Rottloff et al., 2016); *N. albomarginata, N. mirabilis, N. sanguinea* (Rottloff et al., 2016); *N.* × *ventrata* (Lee et al., 2016; Wan Zakaria et al., 2019)
ROS regulation	Cationic peroxidase 1, *Nr*Prx1 Cationic peroxidase 1, Prx1	√ −*	√ √	√ √	*N.* × *ventrata* (Lee et al., 2016; Wan Zakaria et al., 2019); *N. alata* (Hatano and Hamada, 2008); and *N. bicalcarata* (Rottloff et al., 2016)
	Cationic peroxidase 3, *Nr*Prx3	−	−	√	
Secondary metabolism	Cytochrome P450	√	√	−*	
	Isoflavone 2′-hydroxylase	√	−*	√	
	Isoflavone reductase homolog	−*	√	√	
	12-oxophytodienoate reductase 3, OPR3	√	√	√	

^‡^A, *N. ampullaria;* H, Hybrid; R, *N. rafflesiana*; √, Presence; −, Absence. *indicates the presence of transcript in the pitcher tissues but no protein was detected in the pitcher fluids. Refer Supplementary File 3 for details on the transcript ID.

All five reported nepenthesins were found in all species, except for Nep2 that was detected only in *N. rafflesiana* but the Nep2 transcripts were found in all species (Table 3). A recently reported Nep6 discovered in *N.* × *ventrata* (Wan Zakaria et al., 2019) was not detected despite that the sequence can be found in the transcriptomes of *N. rafflesiana* and hybrid (Supplementary File 1). The prolyl endoprotease neprosin Npr1 can be found in all three species while Npr2 was only found in *N. rafflesiana* and hybrid despite the presence of transcript in *N. ampullaria*. We identified a longer sequence (381 amino acids) of Npr2 (c68976/4/1377| m.37184) compared to the partial sequence (304 amino acids) reported by Lee et al. (2016) in *N. rafflesiana* with 81.9% sequence identity (Supplementary Figure 4A). An interesting protease identified in this study is the cysteine-type protease, vignain (c114505/1/1264| m.49694) uniquely found in *N. ampullaria*, which showed 76% sequence identity to partial sequence of peptidase C1 domain-containing protein (GenBank ID: GAV62544.1) present in *Cephalotus follicularis*, a carnivorous pitcher plants from a different plant order. Cathepsin propeptide inhibitor domain (I29) was detected in the sequence, which was found in the N-terminal of several peptidase C1, such as caspase that acts as a propeptide. The cysteine-type protease sequence was compared to a putative protease *Nv*CP1 from *N. ventricosa* reported by Stephenson and Hogan (Athauda et al., 2004) with 49.3% sequence identity (Supplementary Figure 4B). The transcripts of vignain were also found in *N*. *rafflesiana* and the hybrid despite not detected in their pitcher fluids.

This study also found two proteins potentially involved in lipid metabolism, namely non-specific lipid transfer protein GPI-anchored 1 (LTPG1) and lipid phosphate phosphatase 2 (LPP2). These lipid transfer proteins (LTPs) were found in all three *Nepenthes* species. The presence of LTPG1 was reported in *N. alata* and *N. mirabilis* but multiple sequence alignment found limited sequence similarity with 23.2 and 19.8%, respectively, (Supplementary Figure 4C). Two β-1,3-glucanases were detected in *N. rafflesiana* but absent in *N. ampullaria*, while a thaumatin-like protein (TLP) was only found in *N. ampullaria* with also two chitinases possibly involved in polysaccharide metabolism and/or defense response. There were more peroxidases detected in *N. rafflesiana* than *N. ampullaria* with roles in the regulation of reactive oxygen species (ROS).

In this study, we discovered several new proteins in *Nepenthes* pitcher fluids involved in secondary metabolism. Some of these proteins are cytochrome P450, isoflavone 2′-hydroxylase, isoflavone reductase homolog, and 12-oxophytodienoate reductase (jasmonic acid (JA) biosynthesis) potentially involved in secondary metabolism, anti-microbial properties, and stress response.

**FIGURE 5:**
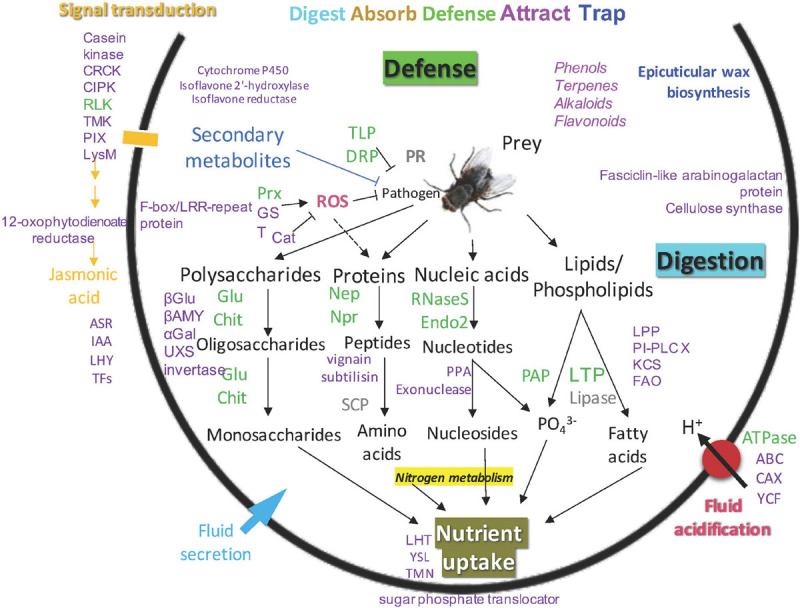
An updated model of carnivory mechanism in *Nepenthes* pitchers, green: common proteins found in pitcher fluids, gray: common proteins not detected in this study, purple: new proteins found in this study, blue: new biological process identified in this study.

Putative functions for identified proteins in the newly opened pitchers of the three *Nepenthes* species are portrayed in the model of *Nepenthes* carnivory mechanism adapted from Lee et al. (2016); (Figure 5). This model supports that the digestive processes can readily occur upon pitcher opening through endogenous hydrolytic proteins even in the absence of prey. Four main types of metabolism identified include polysaccharide, protein, nucleic acid, and lipid digestion. The digestion of prey is likely to be initiated by glucanase and chitinase that digest the cell wall and outer parts of insects, providing nitrogen and phosphate to the plant.

## Discussion

### A Reference Transcriptome for Protein Identification

We have generated full-length transcriptomes for species-specific protein profiling of three *Nepenthes* species during the early stage of pitcher opening to identify endogenous proteins that may contribute to the carnivory traits of *Nepenthes*. The PacBio sequencing of transcriptome from individual species provided a reference of 65,757 predicted protein sequences for protein identification. Previously, Biteau et al. (2013) examined just-opened pitchers of *N. ampullaria* and 12 other *Nepenthes* species using acetone precipitation with SDS-PAGE method followed by matrix-assisted laser desorption/ionization-time of flight (MALDI-TOF) MS analysis. Meanwhile, Rottloff et al. (2016) investigated fluids from closed pitchers of several *Nepenthes* species including *N. rafflesiana* using one-dimensional SDS-PAGE followed by electrospray ionization-tandem MS (ESI-MS/MS) analysis. Lee et al. (2016) first reported proteins in *N.* × *ventrata* pitcher fluids using PIT approach through in-gel and gel-free proteomic analyses based on *N. rafflesiana* transcriptome from Illumina RNA sequencing. More recently, Wan Zakaria et al. (2019) investigated the protein replenishment in the pitcher fluids of *N.* × *ventrata* through species-specific RNA-seq analysis (Wan Zakaria et al., 2016a) and label-free quantitative proteomics (LC–MS/MS) (Wan Zakaria et al., 2018), which led to the discovery of a new nepenthesin-6 (Nep6) found to be replenished after its depletion upon pitcher opening. We also adopted the PIT approach to identify proteins in the pitcher fluids of newly opened pitchers. Due to limited protein content in *Nepenthes* pitcher fluids as previously reported (Buch et al., 2015; Lee et al., 2016), which used pooled samples of up to 1,000 pitcher fluids, we pooled nine samples to yield more concentrated proteins for nanoLC-MS/MS analysis. To our knowledge, this is the first study to analyze the transcriptome and proteome of *N.* × *hookeriana* in relation to its parent species, *N. ampullaria* and *N. rafflesiana*, to compare the pitcher fluid protein compositions related to different dietary habits.

### Proteins Commonly Found in the Pitcher Fluids

A total of 220 proteins were found in pitcher fluids of the three *Nepenthes* species, including proteins known to be involved in the digestive mechanism of *Nepenthes*. Previously, several classes of proteins from *Nepenthes* pitcher fluid had been discovered, such as proteins involved in digestion, pitcher maturation, pathogenesis-related (PR), or defense (Stephenson and Hogan, 2006; Hatano and Hamada, 2008; Buch et al., 2014; Rottloff et al., 2016). Proteome analysis of *N. alata* found six proteins, including three novel PR proteins, namely TLP, β-1,3-glucanase, and β-D-xylosidase, that exhibit anti-microbial properties (Hatano and Hamada, 2012; Buch et al., 2014), which were also found in our study, except for β-D-xylosidase (Table 3). Lee et al. (2016) identified 36 proteins while Wan Zakaria et al. (2019) found 32 proteins from insect-fed and no insect-fed *N.* × *ventrata* pitcher fluids, respectively. Fukushima et al. (2017) focused on well-known secreted proteins (aspartic protease, class III peroxidase, GH18/class III chitinase, GH19/class IV chitinase, β-1,3-glucanase, TLP, purple acid phosphatase, PR-1-like protein, and RNase T2) from four different families of pitchers plants with three independent carnivorous origins, including *N. alata*. They showed convergent evolution of plant carnivory in the amino acid substitutions of some of the conserved digestive enzymes.

Overall, the number of proteins identified from previous studies were much lower than our findings of 94 to 125 identified proteins for individual species with the highest number from *N. rafflesiana* and 25 proteins shared in all three species. This might be due to the differences in datasets and analysis pipelines used for protein identification. Our findings from the functional annotation of *Nepenthes* transcriptomes (Figure 2) and identified proteins from proteomic analyses (Figure 3) revealed that *N.* × *hookeriana* is more similar to *N. rafflesiana* as compared to *N. ampullaria*, which is consistent to findings from the genetic analysis (Yulita and Mansur, 2012) that suggest a greater genetic similarity between the two species than *N. ampullaria*. Since our samples were obtained originally from natural habitat instead of controlled breeding, we cannot exclude the possibility of genetic similarity derived from hybrid backcrossing with *N. rafflesiana*. Nevertheless, a higher number of common proteins between *N.* × *hookeriana* and *N. rafflesiana* observed in protein clustering of transcriptomics and proteomics analyses suggested that a similar set of proteins were secreted during the early stage of pitcher opening in our samples, and both species have similar enzymes for prey digestion.

The 25 proteins found in all three *Nepenthes* species reflect their importance for early processes in newly opened *Nepenthes* pitchers. GO annotations for transcriptome and proteome discovered biological processes and molecular functions of the proteins identified in the pitcher fluids with the lowest significant difference between *N. rafflesiana* and the hybrid. Some of the identified proteins in this study involved four main types of metabolisms, which are the metabolisms of proteins (10), lipids (7), nucleic acids (5), and polysaccharides (5). A high number of proteins involved in the catalytic activity were found with enzymatic roles for digestion, such as the hydrolase activities nepenthesins, purple acid phosphatase, and lipid phosphate phosphatase 2, which were common in all three species (Table 2).

Many of these proteins are found in previous studies (Athauda et al., 2004; Hatano and Hamada, 2012; Buch et al., 2014; Rottloff et al., 2016; Fukushima et al., 2017), which suggested that these endogenous proteins were secreted during pitcher opening in preparation for defense and prey digestion. These proteins include the conserved aspartic proteases, nepenthesins (Nep1-Nep5) that function to digest prey, mainly insects and plant debris by hydrolyzing peptides (Athauda et al., 2004; Lee et al., 2016). Most of these nepenthesins were found in other *Nepenthes* species such as *N.* × *ventrata*, *N. alata*, *N. distillatoria*, and *N. gracilis* (Athauda et al., 2004; Hatano and Hamada, 2008; Nishimura et al., 2014; Rottloff et al., 2016), except the Nep2, which was only found in *N. rafflesiana* with the same similarity to the *Nr*Nep2 sequence found by Lee et al. (2016) in *N.* × *ventrata* pitcher fluids. Nep1 contains carbohydrate moieties and glycosylation sites important for protein stability to prevent denaturation. However, it was not the case for Nep2, as observed in *N. gracilis* (Bariola and Green, 1997), which could explain the probable instability of Nep2 in the pitcher fluids, hence not found in *N. ampullaria* and *N.* × *hookeriana* despite the presence of transcripts (Table 3). Another recently reported nepenthesin in *N.* × *ventrata*, Nep6 (Wan Zakaria et al., 2019), was not detected in any of the species but the sequence was found in the reference transcriptome (c202852/1/1129| m.78569) attributed by *N. rafflesiana*. It is noteworthy that nine out of 10 proteases reported in this study were found in *N. rafflesiana* compared to six in the hybrid and *N. ampullaria*. Furthermore, only Nep4 and CLPX were identified by the “specific” trypsin digestion setting, while majority of other proteases were identified by “semispecific” and “unspecific” digestion settings, suggesting protein self-hydrolysis in the pitcher fluids during protein extraction (Supplementary File 3).

The presence of pathogenesis or defense-related proteins such as TLP, β-1,3-glucanase, and class III and class IV chitinases were not consistent, which suggests differential protein secretion in the three *Nepenthes* species. Both chitinases were secreted in the pitcher fluids of *N. ampullaria* but only one in each of the other species (Table 3). These proteins were found to be prey-induced in *N.* × *ventrata* and *N. alata* (Lee et al., 2016; Rottloff et al., 2016) with proposed contribution to the anti-microbial environment in the pitcher fluids apart from digestion (Hatano and Hamada, 2012; Buch et al., 2013). TLP putatively functions to fight pathogens from the ingested prey, while the glycoside hydrolases (GHs), β-1,3-glucanases, and chitinases function in hydrolyzing polysaccharides, such as the cell walls of pathogens, insects, and leaves (Minic and Jouanin, 2006; Buch et al., 2013).

On the other hand, a nuclease, the S-like ribonuclease (RNaseS) was identified in *N.* × *hookeriana* and *N. rafflesiana*, with high sequence similarity to a similar protein in *N.* × *ventrata*, *N. bicalcarata*, and *N. ventricosa* (Stephenson and Hogan, 2006; Nishimura et al., 2014). In non-carnivorous plants, the protein is useful for self-defense against pathogen attacks from the prey (Bariola and Green, 1997; Sangaev et al., 2011). The expression of RNaseS in carnivorous plants showed tissue-specific constitutive expression in *Drosera* and *Cephalotus* and is prey-induced in *Dionaea* (Okabe et al., 2005; Nishimura et al., 2013). The presence of RNaseS in *N. rafflesiana* and the hybrid could indicate the conservation of ribonuclease activity for anticipated insect prey digestion.

### New Proteins Found in the Pitcher Fluids

In this study, we discovered 21 new pitcher fluid proteins involved in protein regulation (Supplementary File 3). These previously unreported proteins mainly function in protein ubiquitination, such as BTB/POZ domain-containing protein (POB1), E3 ubiquitin-protein ligases, F-box/kelch-repeat protein, and F-box/LRR-repeat protein. This suggests that the turnover of secreted proteins is actively regulated in the pitcher fluids. However, no proteasomal protein was found. Therefore, the half-life of secreted proteins in the pitcher fluids poses an interesting biological question to be addressed in the future.

We also discovered 10 proteins related to signal transduction, which may play roles in regulating gene expression with the 28 detected transcription factors (Figures 4, 5 and Supplementary File 3). It is intriguing to find these proteins in the pitcher fluids, which are expected to be intracellular. Likewise, for the 15 proteins functioning in protein translation or synthesis, such as eukaryotic translation initiation factor 1A (EIF1A), 30S and 60S ribosomal proteins, arginyl-tRNA—protein transferase 1, and valine—tRNA ligase. Similarly, there were 10 proteins related to intracellular trafficking or cytoskeleton, such as actin, armadillo repeat-containing kinesin-like protein, and katanin. Some of these unexpected proteins are reported in the previous studies of *N.* × *ventrata* pitcher fluids (Lee et al., 2016; Wan Zakaria et al., 2019).

The carnivory mechanism of carnivorous plants has been proposed to evolve from the plant defense mechanism through JA signaling (Yilamujiang et al., 2016; Pavlovič and Mithöfer, 2019). It is therefore interesting to discover a 12-oxophytodienoate reductase (OPR3) involved in the biosynthesis of JA and lipid metabolism (Chini et al., 2018) in the pitcher fluids of all three species. It had been reported that JA may induce the proteolytic activity of nepenthesin in *Nepenthes* (Buch et al., 2015). Other phytohormone-related proteins include auxin response proteins (IAA9 and 27), abscisic stress-ripening protein 1 (ASR1), LHY, and WRKY transcription factors, which could play a role in stress response.

Apart from OPR3, we detected several other proteins involved in secondary metabolism, which include the cytochrome P450, isoflavone 2′-hydroxylase, and isoflavone reductase homolog. The cytochrome P450 functions to convert carlatone to carlartonic acid and is involved in flavonoid pathway (Abe et al., 2014); isoflavone 2′-hydroxylase functions in the biosynthesis of isoflavonoid-derived antimicrobial compounds (Akashi et al., 1998); and isoflavone reductase functions in the biosynthetic pathway of isoflavonoid phytoalexin (Cheng et al., 2015). Previous studies identified flavonoids and naphthoquinones in *N. khasiana* (Eilenberg et al., 2006), naphthoquinones (plumbagin and 7-methyl-juglone) in the opened pitcher fluids of *N. ventricosa* (Buch et al., 2013), while dihydronaphthoquinone glucosides rossoliside, plumbaside A, and plumbagin were reported in *N. insignis* (Rischer et al., 2002). These metabolites contain anti-microbial properties that prevent microbial competition for nutrient absorption. Efforts in the profiling of secondary metabolites from *Nepenthes* pitchers and their bioactivity are on-going (Rosli et al., 2017, 2018; Dávila-Lara et al., 2020). Meanwhile, genes involved in the biosynthesis of secondary metabolites such as phenylpropanoids, sesquiterpenoids, and triterpenoids in *N. ampullaria* were found to be influenced by endogenous protein depletion (Goh et al., 2020). Proteins involved in secondary metabolism were also reported to be important for response against environmental stress such as pathogen attack that led to the synthesis of secondary metabolites from different pathways (Chini et al., 2018; Goh et al., 2020). Further studies are needed to ascertain the roles of these proteins in secondary metabolism and stress response. Multi-omics integration will help elucidate the genes or enzymes involved in the biosynthesis pathways of secondary metabolites important for pitcher physiology.

Despite the discovery of many new proteins, most of them are expected to be functional intracellularly, such as OPR3 in the peroxisomes, transcription factors in the nucleus, and the membrane-localized transporters. Since our experimental design is based on species-specific transcriptomes using newly opened pitchers without prey, it is unlikely that these proteins are contaminants from the microbes or insects. However, we cannot exclude the possibility that these proteins could be attributed by microbial symbionts of the pitcher plants that could be present even in closed pitchers, although the fluids are unsuitable for microbial growth (Buch et al., 2013). The significance of these seemingly intracellular proteins in the pitcher fluids warrants further studies. It is noteworthy that the discovery of extracellular OPR3 corroborates the presence of jasmonyl-isoleucine (JA-Ile) in the digestive fluid (Yilamujiang et al., 2016). This suggests the possibility of the biosynthesis of phytohormones or secondary metabolites extracellularly.

On the other hand, there is no strong evidence in this study to suggest an adaptive evolution of *N. ampullaria* with novel enzymes for digesting leaf litter, which has been hypothesized to depend on infauna of the pitcher fluids (Moran et al., 2003; Moran and Clarke, 2010). This is consistent with the findings that pitcher fluids of *N. ampullaria* are heavily populated with aquatic organisms (Cresswell, 1998), perhaps due to the less acidic pitcher fluids compared to other *Nepenthes* species at a trade-off of hydrolytic enzymes functioning at suboptimal pH (Saganová et al., 2018). Nonetheless, some of the unique endogenous proteins discovered in *N. ampullaria* could potentially contribute to nutrient sequestration, for example, a cysteine-type peptidase vignain, an alpha-galactosidase, a beta-glucosidase, a cellulose synthase A (CESA), and a catalase (Figure 3). Apart from these unique enzymes, the finding that both prey-induced chitinases (Chit1 and Chit3) were found in the newly opened pitchers suggests differential secretion of proteins in *N. ampullaria* could contribute to its success in being an omnivore to derive nutrients from both insects and leaf litter. However, this remains speculative without functional validation through genetic transformation or transfection, which unfortunately is still unavailable.

## Conclusion

The comparison of protein content in pitcher fluids of three *Nepenthes* species through transcriptomic and proteomic analyses revealed distinct profiles of secreted proteins, especially hydrolytic enzymes and defense-related proteins. Despite no evidence of novel enzymes for leaf litter digestion in *N. ampullaria*, this study provides information on the molecular compositions of individual *Nepenthes* species with differential secretion of endogenous proteins apart from the distinct morphological traits between the parent species and hybrid that reflect inter-species diversity. Furthermore, many interesting biological questions that are raised on the functions of new proteins discovered in this study manifest wonders on the molecular physiology of secreted proteins in the pitcher fluids to be elucidated in future studies.

## Data Availability Statement

The datasets presented in this study can be found in online repositories. The names of the repository/repositories and accession number(s) can be found in the article/Supplementary Material.

## Author Contributions

MZ, WA, and H-HG designed the experiments. MZ performed the experiments and analyzed the data. H-HG performed funding project administration and supervision. NA and TT assisted in data organization and discussion. MZ, NA, TT, WA, and H-HG wrote and edited the manuscript. All authors contributed to the article and approved the submitted version.

## Conflict of Interest

The authors declare that the research was conducted in the absence of any commercial or financial relationships that could be construed as a potential conflict of interest.

## Footnotes

1 https://doi.org/10.6084/m9.figshare.13270241.v1

2 http://eggnog-mapper.embl.de/

## Supplementary Material

Supplementary Figure 1 Overview of the transcriptomics and proteomics studies of *Nepenthes* pitcher fluids.

Supplementary Figure 2 BLASTN analysis of consensus isoform sequences. **(A)** BLASTN searches of individual transcriptomes against the reference transcriptome. BLASTN analysis of hybrid transcriptome against the parent transcriptomes showing **(B)** the percentage of hybrid sequences that found hits and **(C)** the percentage of parent sequences that found hits with the hybrid in reciprocal searches.

Supplementary Figure 3 SDS-PAGE and silver staining analysis of pitcher fluid protein extraction and processing. **(A–C)** SDS-PAGE gel results for 20 μL aliquots after filtration (left) and after concentration (right). **(A)** *N. ampullaria* [amp]. **(B)** *N. rafflesiana* [raff]. **(C)** *N.* × *hookeriana* [hook]. Individual lanes represent the nine biological replicates. **(D)** SDS-PAGE gel results of pooled samples after solid phase extraction (left) and peptide digestion (right).

Supplementary Figure 4 Multiple sequence alignment analysis of selected sequences found in proteomic analysis. **(A)** Pairwise sequence alignment of c68976/4/1377| m.37184 transcript (top) and truncated Neprosin-2 transcript (bottom) from *N. rafflesiana* as reported by Lee et al. (2016). **(B)** Pairwise sequence alignment of c114505/1/1264 m.49694 (top) and *Nv*CP1 (bottom), with a sequence identity of 49.34%. **(c)** Sequence alignment of c171049/1/1434 m.73671 and LTP sequences from *N. alata* and *N. mirabilis*.

Supplementary Table 1 Statistics of transcriptome obtained from PacBio sequencing using the RS_IsoSeq protocol. The analysis had been performed for independently individual species. Reference dataset is generated from the combined analysis of all three transcriptomes.

Supplementary Table 2 WEGO analysis of the gene ontology (GO) annotation.

Supplementary Table 3 Summary of KO analysis.

Supplementary Table 4 OrthoVenn cluster analysis of predicted protein sequences.

Supplementary File 1 Functional annotation of transcriptomes.

Supplementary File 2 GO and KO analysis.

Supplementary File 3 Proteomics analysis.
