# Supplementary material for: Transcriptomic and Proteomic Analyses of Nepenthes ampullaria and Nepenthes rafflesiana Reveal Parental Molecular Expression in the Pitchers of Their Hybrid, Nepenthes × hookeriana
Source: Front Plant Sci. 2021 Jan 20;11:625507. doi: 10.3389/fpls.2020.625507 (PMC7855304; doi:10.3389/fpls.2020.625507)
Supplement: Supplementary Figure 1 — Overview of the transcriptomics and proteomics studies of Nepenthes pitcher fluids. [file Data_Sheet_1.PDF]

Supplementary Material

Figure S1

Proteomics Informed by Transcriptomics (PIT) Approach

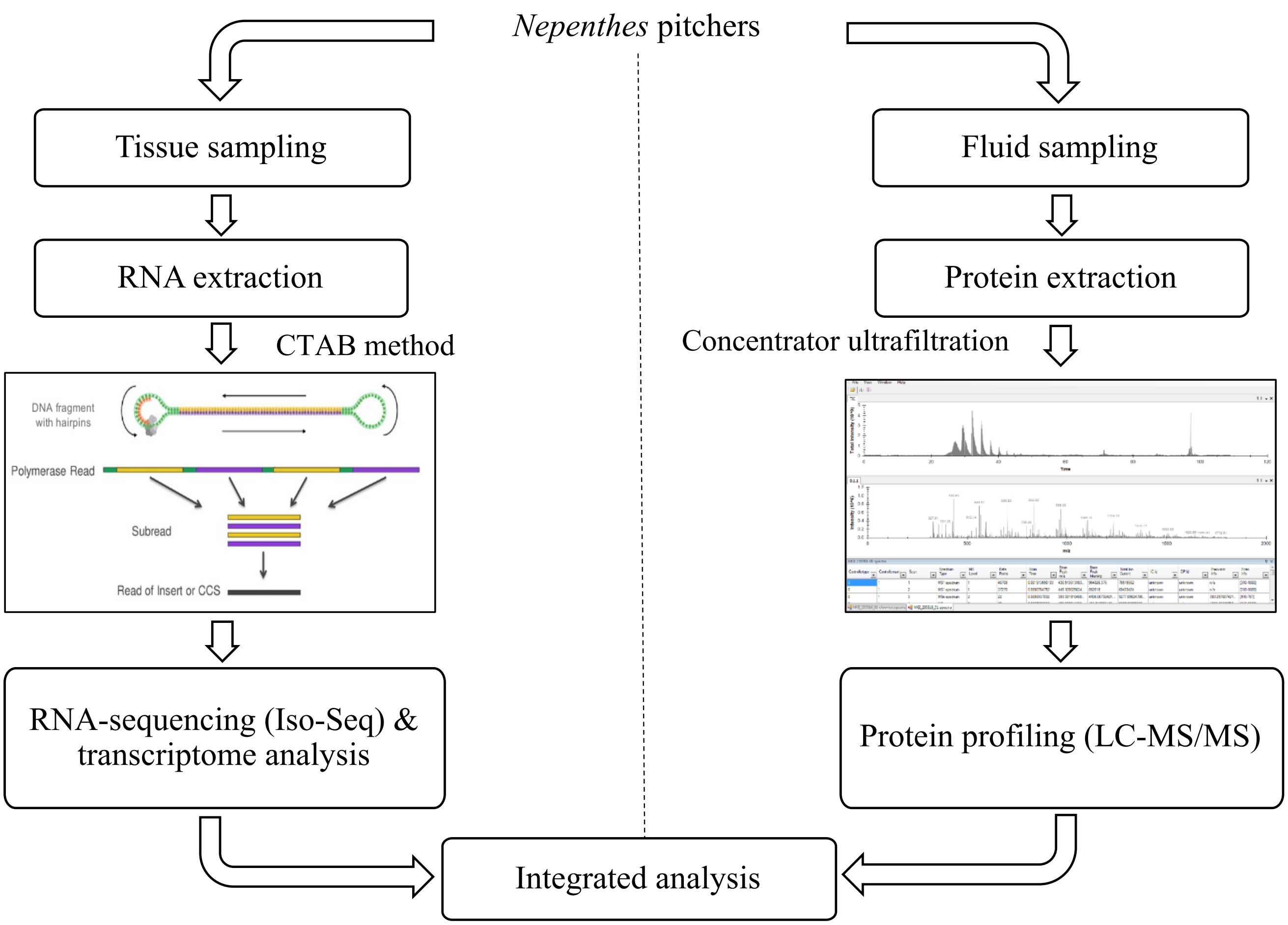

Figure S1. Overview of the transcriptomics and proteomics studies of *Nepenthes* pitcher fluids.

Figure S2

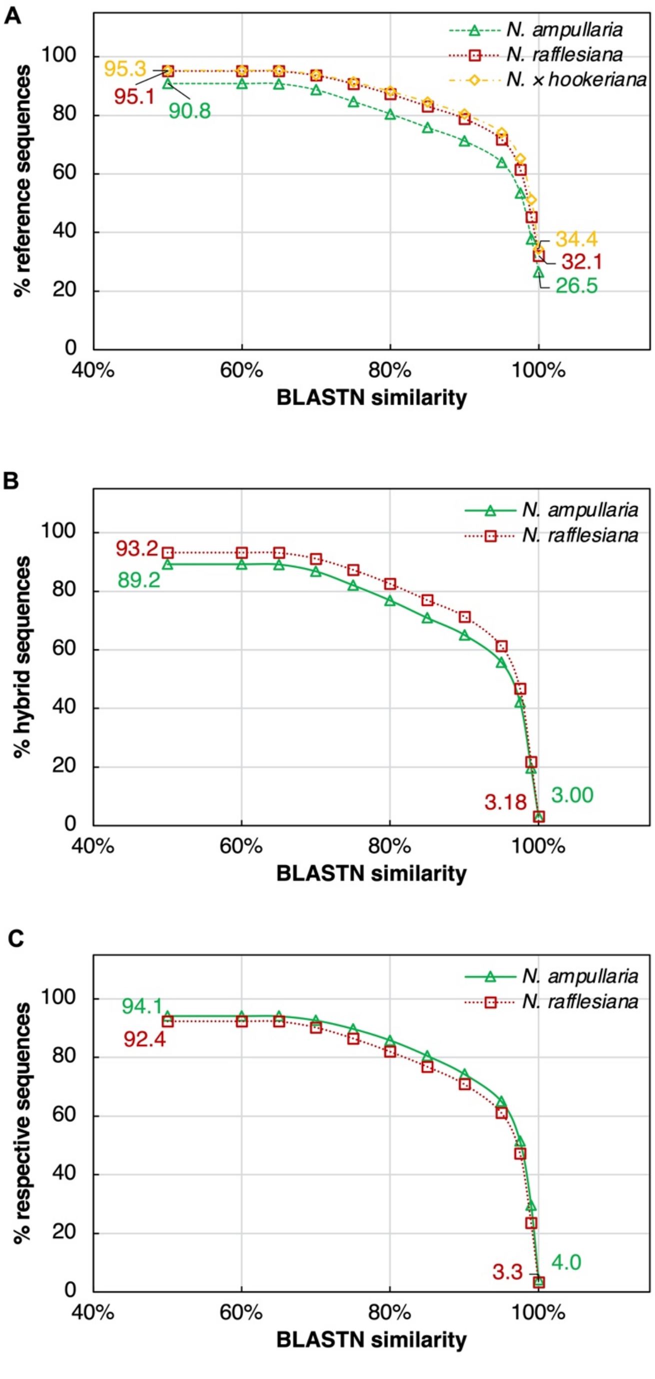

Figure S2. BLASTN analysis of consensus isoform sequences. (A) BLASTN searches of individual transcriptomes against the reference transcriptome. BLASTN analysis of hybrid transcriptome against the parent transcriptomes showing (B) the percentage of hybrid sequences which found hits and (C) the percentage of parent sequences which found hits with the hybrid in reciprocal searches. Percentage values were calculated based on the total sequences for respective transcriptomes at different thresholds of BLASTN similarity.

Figure S3

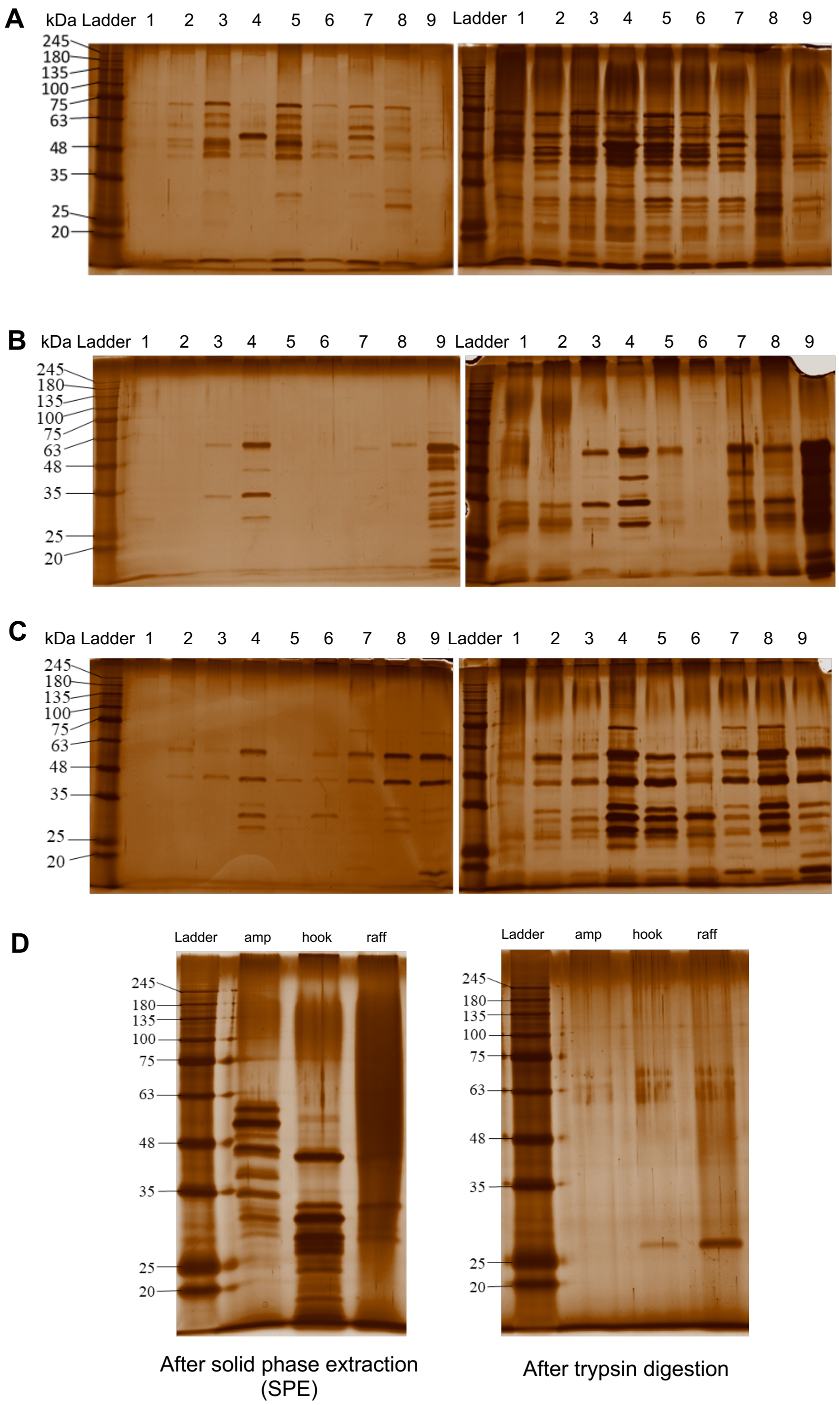

Figure S3. SDS-PAGE and silver staining analysis of pitcher fluid protein extraction and processing. (**A**-**C**) SDS-PAGE gel results for 20 µL aliquots after filtration (left) and after concentration (right). (**A**) *N. ampullaria* [amp]. (**B**) *N. rafflesiana* [raff]. (**C**) *N.* × *hookeriana* [hook]. Individual lanes represent the nine biological replicates. (**D**) SDS-PAGE gel results of pooled samples after solid phase extraction (left) and peptide digestion (right).

Figure S4

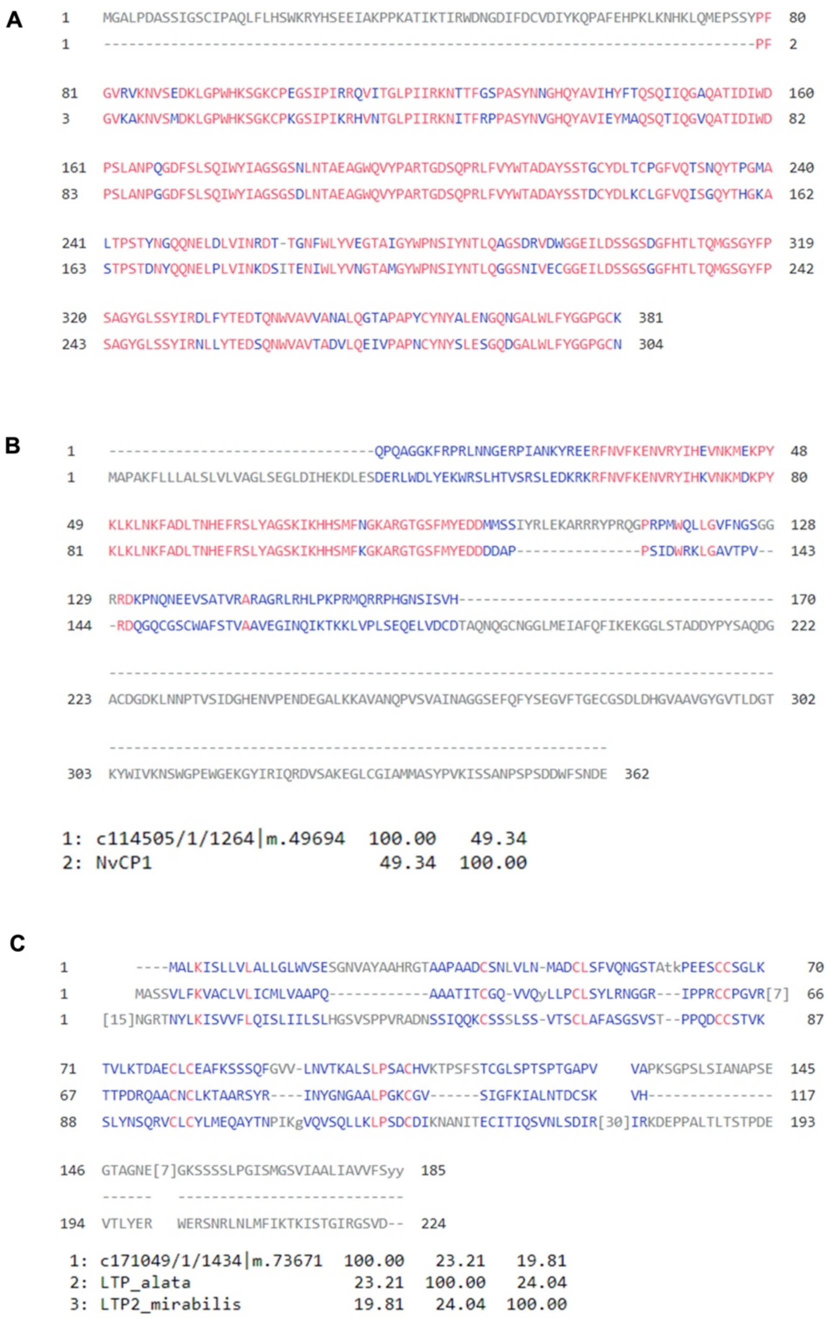

Figure S4. Multiple sequence alignment analysis of selected sequences found in proteomic analysis. (**A**) Pairwise sequence alignment of c68976/4/1377|m.37184 transcript (top) and truncated Neprosin-2 transcript (bottom) from *N. rafflesiana* as reported by Lee et al. (2016). (**B**) Pairwise sequence alignment of c114505/1/1264 m.49694 (top) and NvCP1 (bottom), with sequence identity of 49.34%. (**C**) Sequence alignment of c171049/1/1434 m.73671 and LTP sequences from *N. alata* and *N. mirabilis*.

Table S1. Statistics of transcriptome obtained from PacBio sequencing using the RS_IsoSeq protocol. The analysis had been performed for independently individual species. Reference dataset is generated from the combined analysis of all three transcriptomes.

**Attribute**	**Species**			**Reference**
	N. ampullaria	***N. rafflesiana***	***N. × hookeriana***	
Read bases of insert	154,845,182	166,851,165	164,740,830	486,437,177
Mean read length of insert (bp)	1,792	1,852	1,910	1,851
Mean read quality of insert	93.6%	93.7%	93.8%	93.7%
Mean no. of passes	9.23	9.05	9.29	9.00
No. of reads of insert (ROI)	86,407	90,076	86,246	262,729
No. of 5’ reads	58,433	58,647	60,760	177,840
No. of 3’ reads	59,448	63,228	63,815	186,491
No. of poly-A reads	49,162	61,347	61,339	171,848
No. of filtered short reads	5,834	5,631	3,613	15,078
No. of full-length (FL) reads	48,470	49,002	51,606	149,078
No. of non-FL reads	32,103	35,443	31,027	98,573
No. of FL non-chimeric reads	48,147	48,552	51,265	147,964
Average FL non-chimeric read length (bp)	1,590	1,623	1,668	1,628
No. of consensus isoforms	26,130	30,558	33,279	80,791
Average length of consensus isoforms (bp)	1,625	1,680	1,722	1,692

Table S2. WEGO analysis of the gene ontology (GO) annotation.

**Description**	**Number**
Total GO terms	495
Biological Process	310
Molecular Function	100
Cellular Component	85
**Number of GO terms with significance difference (*P*<0.05)**	
N. ampullaria vs. N. rafflesiana	76 (15.4%)
N. × hookeriana vs. N. ampullaria	61 (12.3%)
N. × hookeriana vs. N. rafflesiana	43 (8.7%)

Table S3. Summary of KO analysis.

Number	Species			Reference
	N. ampullaria	***N. rafflesiana***	***N. × hookeriana***	
KO	2,432	2,846	2,663	3,419
KEGG pathway	395	398	396	400
Unique KEGG pathway	2	1	1	0

Table S4. OrthoVenn cluster analysis of predicted protein sequences.

Attribute	Species			Reference
	N. ampullaria	***N. rafflesiana***	***N. × hookeriana***	
Number of consensus isoforms	14,523	19,683	22,192	48,663
Number of predicted peptides	19,463	26,677	30,096	65,757
Clusters	5,709	6,878	7,291	
Singletons	12,466	17,561	19,707	
**Comparison**	**Number of shared clusters**		**% (hybrid)**	
N. ampullaria vs. N. rafflesiana	4,355		-	
N. ampullaria vs. N. × hookeriana	4,656		63.9	
N. rafflesiana vs. N. × hookeriana	5,565		76.3	

**Singletons are proteins not in any cluster*
